# Supplementary material for: Location of Trigger Points in a Group of Police Working Dogs: A Preliminary Study
Source: Animals (Basel). 2023 Sep 7;13(18):2836. doi: 10.3390/ani13182836 (PMC10526025; doi:10.3390/ani13182836)
Supplement: Supplementary file 1 [file animals-13-02836-s001.zip › animals-2506159-supplementary.pdf]

animals

MDPI

Communication

Location of Trigger Points in a Group of Police Working Dogs: A Preliminary Study

Maira Rezende Formenton [1,*], Karine Portier [2,3], Beatriz Ribeiro Gaspar [1], Lisa Gauthier [2], Lin Tchia Yeng [4] and Denise Tabacchi Fantoni [1]

1 School of Veterinary Medicine and Animal Science, University of São Paulo, São Paulo 05508-270, Brazil; beatriz.gaspar@usp.br (B.R.G.); dfantoni@usp.br (D.T.F.)
2 VetAgro Sup (Campus Vétérinaire), Centre de Recherche et de Formation en Algologie Comparée (CREFAC), University of Lyon, 69280 Marcy l'Etoile, France; karine.portier@vetagro-sup.fr (K.P.); lisagauthier97@gmail.com (L.G.)
3 Centre National de la Recherche Scientifique (CNRS), Institut National de la Santé et de la Recherche Médicale (INSERM), Centre de Recherche en Neurosciences de Lyon (CRNL), University of Lyon, U1028 UMR 5292, Trajectoires, 69500 Bron, France
4 School of Medicine, Institute of Orthopedics and Traumatology, University of São Paulo, São Paulo 05403-010, Brazil; lintyeng@uol.com.br
* Correspondence: mairaformenton@gmail.com; Tel.: +(55)-11-98567-5756

Link for Video S1:

https://drive.google.com/drive/folders/1KFWKqQCTAzrzdBA-jl7NUCwZY5mvLTOo?usp=sharin
g

Table S1: Dogs individual information.

Number	Age (Years)	Sex (F/M)	Weight (Kg)	Breed	Work
1	7	F	36	Dutch Shepperd	Drug Sniffing
2	3	F	25	German Shepperd	Drug Sniffing
3	5	M	30,1	Mallinois Belgian	Drug Sniffing
4	5	M	33,3	Mallinois Belgian	Drug Sniffing
5	7	M	31	Mallinois Belgian	Search And Rescue
6	3	F	27,2	German Shepperd	Drug Sniffing, Search And Rescue
7	8	M	29,5	Mallinois Belgian	Explosive Detection
8	4	M	34,1	Labrador Retriever	Explosive Detection
9	8	M	32,9	Mallinois Belgian	Drug Sniffing, Guarding
10	3	M	29	German Shepperd	Drug Sniffing, Guarding
11	6	F	28	Mallinois Belgian	Drug Sniffing, Guarding
12	6	F	20	Mallinois Belgian	Drug Sniffing

Table S2: Dogs individual trigger point location by each evaluator.

DOG	TP LOCATION	Side	EVALUATOR 1	EVALUATOR 2
1	LATISSIMUS DORSI 2	RIGHT	x	x
1	LONGISSIMUS LUMBORUM PART 1	RIGHT	x	x
1	TRICEPS BRACHII 1	RIGHT	x	x
1	QUADRICEPS FEMORIS 1	RIGHT	x	x
1	BICEPS FEMORIS	RIGHT	x	
1	TRAPEZIUS PARS THORACICA	RIGHT		x
1				
1	DELTOIDEUS	LEFT	x	
1	SARTORIUS	LEFT	x	
1	GLUTEUS MEDIUS	LEFT	x	
1	LATISSIMUS DORSI 4	LEFT		x
1	LONGISSIMUS LUMBORUM PART 1	LEFT		x
1	BICEPS FEMORIS	LEFT		x
1	PECTINEUS	LEFT		x
2	BICEPS FEMORIS	RIGHT	x	x
2	LONGISSIMUS LUMBORUM PART 2	RIGHT		x
2				
2	BICEPS FEMORIS	LEFT	x	
2	TRAPEZIUS PARS THORACICA	LEFT	x	
3	LATISSIMUS DORSI 2	RIGHT	x	x
3	SARTORIUS	RIGHT	x	
3	QUADRICEPS FEMORIS 2	RIGHT		x
3	PECTINEUS	RIGHT		x
3				
3	TRAPEZIUS PARS THORACICA	LEFT	x	
3	LONGISSIMUS LUMBORUM PART 2	LEFT		x
4	LATISSIMUS DORSI 2	RIGHT	x	x
4	QUADRICEPS FEMORIS 2	RIGHT	x	x
4	TRICEPS BRACHII 1	RIGHT		x
4	LONGISSIMUS LUMBORUM PART 2	RIGHT		x
4	GLUTEUS MEDIUS	RIGHT		x
4	PECTINEUS	RIGHT		x
4				
4	LATISSIMUS DORSI 2	LEFT	x	

4	QUADRICEPS FEMORIS 1	LEFT	x	x
4	LONGISSIMUS LUMBORUM PART 2	LEFT		x
**DOG**	**TP LOCATION**	**Side**	**EVALUATOR 1**	**EVALUATOR 2**
5	QUADRICEPS FEMORIS 2	RIGHT	x	x
5	PECTINEUS	RIGHT	x	
5	LATISSIMUS DORSI 3	RIGHT		x
5	GLUTEUS MEDIUS	RIGHT		x
5				
5	GLUTEUS MEDIUS	LEFT	x	
5	QUADRICEPS FEMORIS 2	LEFT	x	
5	BICEPS FEMORALIS	LEFT	x	
5	LATISSIMUS DORSI 2	LEFT		x
6	QUADRICEPS FEMORIS 2	RIGHT	x	x
6	LONGISSIMUS LUMBORUM PART 1	RIGHT		x
7	LATISSIMUS DORSI 3	RIGHT	x	x
7	QUADRICEPS FEMORIS 1	RIGHT	x	
7	GLUTEUS MEDIUS	RIGHT		x
7				
7	LATISSIMUS DORSI 2	LEFT	x	x
7	GLUTEUS MEDIUS	LEFT	x	
8	DELTOIDEUS	RIGHT	x	
8	SARTORIUS	RIGHT	x	
8	LONGISSIMUS LUMBORUM PART 2	RIGHT		x
8	LONGISSIMUS THORACICAE PART 2	RIGHT		x
8	QUADRICEPS FEMORIS 1	RIGHT		x
8	PECTINEUS	RIGHT		x
8				
8	DELTOIDEUS	LEFT	x	
8	SARTORIUS	LEFT	x	
8	LONGISSIMUS LUMBORUM PART 2	LEFT		x
8	QUADRICEPS FEMORIS 1	LEFT		x
8	PECTINEUS	LEFT		x
9	LONGISSIMUS LUMBORUM PART 2	RIGHT		x
10	LATISSIMUS DORSI 2	RIGHT	x	x
10	SARTORIUS	RIGHT	x	

10	TRICEPS BRACHII 2	RIGHT		x
10	LONGISSIMUS LUMBORUM PART 2	RIGHT		x
10	LATISSIMUS DORSI 1	LEFT	x	
19	SARTORIUS	LEFT	x	
**NAME**	**TP LOCATION**	**Side**	**EVALUATOR 1**	**EVALUATOR 2**
11	LONGISSIMUS THORACICAE PART 2	RIGHT	x	
11	LATISSIMUS DORSI 5	RIGHT		x
11	SARTORIUS	RIGHT		x
11				
11	LONGISSIMUS THORACICAE PART 2	LEFT	x	
11	LATISSIMUS DORSI 4	LEFT		x
12	LONGISSIMUS THORACICAE PART 2	RIGHT	x	x
12	LONGISSIMUS THORACICAE PART 2	LEFT	x	
